# Bone Mesenchymal Stem Cells Promote Extracellular Matrix Remodeling of Degenerated Nucleus Pulposus Cells *via* the miR-101-3p/EIF4G2 Axis

**DOI:** 10.3389/fbioe.2021.642502

**Published:** 2021-08-27

**Authors:** Zeng Wang, Xiaolin Ding, Feifei Cao, Xishan Zhang, Jingguo Wu

**Affiliations:** ^1^Department of Orthopedics, The Second Affiliated Hospital of Shandong First Medical University, Tai’an, China; ^2^Department of Out-Patient, Tai’an Central Hospital Branch, Tai’an, China

**Keywords:** intervertebral disc degeneration, miR-101-3p, EIF4G2, bone mesenchymal stem cells, nucleus pulposus cells, extracellular matrix remodeling, Collagen-I, Collagen-II

## Abstract

The etiology of lumbocrural pain is tightly concerned with intervertebral disk degeneration (IDD). Bone mesenchymal stem cell (BMSC)-based therapy bears potentials for IDD treatment. The properties of microRNA (miRNA)-modified BMSCs may be altered. This study investigated the role and mechanism of BMSCs promoting extracellular matrix (ECM) remodeling of degenerated nucleus pulposus cells (NPCs) *via* the miR-101-3p/EIF4G2 axis. NPCs were collected from patients with IDD and lumbar vertebral fracture (LVF). The expressions of miR-101-3p and ECM-related proteins, Collagen-I (Col-I) and Collagen-II (Col-II), were detected using the reverse transcription-quantitative polymerase chain reaction. The expressions of Col-I and Col-II, major non-collagenous component Aggrecan, and major catabolic factor Matrix metalloproteinase-13 (MMP-13) were detected using Western blotting. BMSCs were cocultured with degenerated NPCs from patients with IDD. Viability and apoptosis of NPCs were measured using 3-(4,5-dimethylthiazol-2-yl)-2,5-diphenyltetrazolium bromide assay and flow cytometry. After the degenerated NPCs were transfected with the miR-101-3p inhibitor, the expressions of ECM-related proteins, cell viability, and apoptosis were detected. The targeting relationship between miR-101-3p and EIF4G2 was verified. Functional rescue experiments verified the effects of miR-101-3p and EIF4G2 on ECM remodeling of NPCs. Compared with the NPCs of patients with LVF, the degenerated NPCs of patients with IDD showed downregulated miR-101-3p, Col-II, and Aggrecan expressions and upregulated MMP-13 and Col-I expressions. BMSCs increased the expressions of miR-101-3p, Aggrecan, and Col-II, and decreased the expressions of MMP-13 and Col-I in degenerated NPCs. BMSCs enhanced NPC viability and repressed apoptosis. Downregulation of miR-101-3p suppressed the promoting effect of BMSCs on ECM remodeling. miR-101-3p targeted EIF4G2. Downregulation of EIF4G2 reversed the inhibiting effect of the miR-101-3p inhibitor on ECM remodeling. In conclusion, BMSCs increased the miR-101-3p expression in degenerated NPCs to target EIF4G2, thus promoting the ECM remodeling of NPCs.

## Introduction

Approximately 84% of the population worldwide experiences low back pain in their lifetime, of which 10% develops into chronic disability, thus gravely affecting the quality of life and bringing huge financial burdens to families and society (Gore et al., 2012). Intervertebral disk degeneration (IDD) represents an extensively identified contributor to low back pain (Risbud and Shapiro, 2014). The intervertebral disk (IVD) is a complicated structure consisting of three anatomical substructures, namely, nucleus pulposus (NP), annulus fibrosus (AF), and cartilaginous endplates, which are essential to maintain the normal function of a disk (Qu et al., 2018). IDD is featured by the reduction of NP and degradation of proteoglycan, Aggrecan, and collagen in the extracellular matrix (ECM), which disrupts the homeostasis of NP and shifts IVD maintenance toward degeneration and catabolism (Wang et al., 2015). The current clinical interventions for IDD mainly include conservative medication and surgery (spinal fusion or total disk replacement); nevertheless, these therapeutic approaches only temporarily relieve the pain symptoms, instead of providing a permanent cure (Konovalov et al., 2016; Zhu et al., 2019). Hence, developing novel and potent therapies for patients with IDD remains an urgent issue to be solved.

Recently, biological therapeutic approaches such as stem cell transplantation for reversing IDD progression have aroused wide interest (Cai et al., 2019). Bone mesenchymal stem cells (BMSCs) have been extensively applied in transplantation research due to their considerable advantages such as high activity, low immunogenicity, and strong differentiation potential (Zhu et al., 2017; Gan et al., 2018). BMSC transplantation possesses the ability to facilitate IVD regeneration in the rabbit and pig models (Cai et al., 2015; Barczewska et al., 2018). BMSCs can enhance the synthesis of remaining ECM in NP and AF tissues and repress the expression of inflammatory mediators and the activity of matrix-degrading enzymes, thus maintaining the IVD structure (Hu et al., 2017). After BMSCs injection, the proteoglycan gene expression is upregulated, and ECM is gradually accumulated in NP (Yang et al., 2009). At present, the mechanism of BMSCs promoting ECM remodeling in IDD needs further exploration.

MicroRNAs (miRNAs) are a class of small non-coding RNA molecules consisting of 18–23 nucleotides, which exert influences on posttranscriptional modulation of gene expression (Bartel, 2009; Shioya et al., 2010). Aberrant miRNA expression is implicated in the pathological processes of IDD, including NP cell (NPC) apoptosis, ECM degradation, and inflammatory response (Wang et al., 2015). The matured miR-101-3p is generated from miR-101-1 and miR-101-2 (the precursor miR) (Li et al., 2017b). miR-101-3p commonly acts as a tumor suppressor in human malignancies such as breast cancer (Li et al., 2017b) and hepatocellular carcinoma (Li et al., 2017a). Gao Z.X. et al. (2020) have revealed that miR-101-3p expression in degenerated NPCs is significantly lower than that in normal cells, and SNHG6 can suppress proliferation and promote apoptosis of NPCs by downregulating miR-101-3p expression and thus promote the occurrence of IDD. Whether miR-101-3p is implicated in the effect of BMSCs on ECM remodeling in IDD remains unknown. This study investigated the role and mechanism of BMSCs promoting ECM remodeling of degenerated NPCs *via* the miR-101-3p/EIF4G2 axis, which shall shed new insight into BMSC transplantation in the treatment of IDD.

## Materials and Methods

### Ethics Statement

This study was approved by the Ethical Committee of Affiliated Hospital of Shandong First Medical University (No. 2016-161). Informed consent was signed by each eligible participant.

### Nucleus Pulposus Tissue Collection

The intervertebral disk tissue samples were collected from 14 patients with IDD-related low back pain (6 females and 8 males) who underwent surgery in Affiliated Hospital of Shandong First Medical University from March 2016 to June 2019 and 6 patients with lumbar vertebral fracture (LVF) without a history of low back pain (3 females and 3 males). Based on the Pfirrmann classification system, preoperative magnetic resonance imaging was performed for grading (Pfirrmann et al., 2001). The IDD samples were graded IV and V, and LVF samples were graded I and II.

### Cell Culture

According to the previous study, NPCs were isolated and cultured (Gao Z.X. et al., 2020; Zhang et al., 2020). NP tissues were isolated from IVD tissues under the stereoscopic microscope. After washing with phosphate-buffered saline (PBS), NP tissues were sliced into small pieces and detached with 0.5% type II collagenase (Roche Diagnostics, Indianapolis, Indiana, United States) for 2 h. The tissue digestive solution was filtered through a cell filter, and the filtrate was collected and centrifuged at 100 *g* for 5 min. The supernatant was removed, and the precipitate was added into the F12 basic medium containing 10% of fetal bovine serum (FBS) and 100 U/ml of penicillin–streptomycin. The obtained cell suspension was transferred into the culture bottle with a density of about 1 × 10^5^/ml in a humid incubator at 37°C with 5% CO_2_. On days 5–7, when the cells completely adhered to the wall, the culture medium was changed for the first time and then changed every 2–3 days. After more than 80% of the cells were covered in the bottom of the flask, the cells were detached with 0.25% of trypsin and passaged at a ratio of 1:2. The cells of the third generation were used in this study. Protein and mRNA levels of MMP-13, Collagen-I (Col-I), Collagen-II (Col-II), and Aggrecan, the specific indicators for degenerative and normal NPCs, were analyzed.

Bone mesenchymal stem cells (BMSCs) and 293T cells were purchased from American Type Culture Collection (Manassas, VA, United States) and cultured in Dulbecco’s modified Eagle’s medium containing 10% of FBS and 100 U/ml of penicillin-streptomycin at 37°C with 5% of CO_2_. NPCs and BMSCs at passage 5 were used for subsequent experiments.

### Cell Transfection

The miR-101-3p inhibitor, miR-101-3p mimic, and their corresponding negative control (NC) were purchased from RiboBio (Guangzhou, China); EIF4G2-siRNA and its corresponding NC were purchased from Genechem Co., Ltd. (Shanghai, China). NPCs in the logarithmic phase were seeded into the 6-well plates (2 × 10^5^ cells/well) and cultured for 24 h. Then, cell transfection was performed using Lipofectamine 2000 (siRNA 10–100 nM and miRNA mimic 50 nM). The subsequent experiments were conducted after 48 h.

### Coculture of NPCs and BMSCs

A 6-well Transwell system (0.4 μm pore size membrane; Corning, NY, United States) was used to evaluate the effect of BMSCs on NPCs. NPCs (1 × 10^5^) were seeded into the basolateral chamber of Transwell plates, and BMSCs (1 × 10^5^) were spread on the apical chamber. After 24 h, NPCs were harvested for further experiments. Specifically, NPC suspension in the basolateral chamber of a Transwell plate of the coculture system was collected into the centrifuge tube. The adherent cells were washed with PBS once, and then the cells were detached with appropriate amounts of trypsin solution. The adherent cells were incubated at room temperature for 5 min until they can be gently blown off. Then, the trypsin solution was sucked out, resuspended in PBS, and recovered to the cell suspension collected in the previous step. After centrifugation at 1,000 *g* for 5 min, the supernatant was discarded and the cells were collected. The cells were gently suspended with PBS and counted.

### Western Blotting

After the coculture of NPCs and BMSCs, NPCs (1 × 10^7^) were collected for the Western blotting analysis. The protein was extracted using the cell lysis buffer (Beyotime, Shanghai, China), and the bicinchoninic acid protein assay kit (Beyotime) was used for protein quantification. The protein was separated by 10% of SDS-PAGE (Beyotime) and transferred onto polyvinylidene fluoride membranes (Millipore, Billerica, MA, United States). The membranes were blocked with PBS buffer containing 5% of skimmed milk for 2 h and then incubated with the primary antibodies at 4°C overnight. Later, the membranes were cultured with the secondary antibody for 2 h. The protein bands were detected using the enhanced chemiluminescence kit (Thermo Fisher Scientific Inc., Waltham, MA, United States) and quantitatively analyzed using Image J software. The antibodies MMP-13 (1:1,000, ab39012), Col-I (1:1,000, ab34710), Col-II (1:500, ab34712), Aggrecan (1:1,000, ab36861), and GAPDH (1:5,000, ab18160) were purchased from Abcam (Cambridge, MA, United States).

### Reverse Transcription-Quantitative Polymerase Chain Reaction

After the coculture of NPCs and BMSCs, NPCs (2 × 10^6^) were collected using the reverse transcription-quantitative polymerase chain reaction (RT-qPCR). The total RNA was isolated from cells using TRIzol reagent (Invitrogen Inc., Carlsbad, CA, United States), purified using DNase, and reverse transcribed into cDNA using reverse transcription kit (Takara Bio Inc., Kyoto, Japan). RT-qPCR was performed on ABI 7500 (ABI, Foster City, CA, United States) using SYBR Premix Ex Taq (Takara, Dalian, China). The relative expression of genes was calculated by using the 2^–ΔΔ*CT*^ method, with GAPDH and U6 as internal references. Primer sequences are illustrated in Table 1.

**TABLE 1:** Primer sequences for RT-qPCR.

Name of primer	Sequences
miR-101-3p-F	GCCGAGTACAGTACTGTGA
miR-101-3p-R	CTCAACTGGTGTCGTGGA
EIF4G2-F	GTGGAGAGTGCGATTGCAGAA
EIF4G2-R	TCTTTAGTCAGCTTCTTCCTC
U6-F	CTCGCTTCGGCAGCACATATACT
U6-R	ATTTGCGTGTCATCCTTGCGCA
GAPDH-F	GAACGGGAAGCTCACTGG
GAPDH-R	GAACGGGAAGCTCACTGG
Col-I-F	TGACCTCAAGATGTGC
Col-I-R	ACCAGACATGCCTCTTGTCC
Col-II-F	GGTGACTACTGGATAGAC
Col-II-R	TGAAGTGGAAGCCGCCA

### 3-(4,5-Dimethylthiazol-2-yl)-2,5-Diphenyltetrazolium Bromide Assay

The cells were seeded into 96-well plates (5,000 cells/well) and cultured for 48 h. Then, the cells were cultured with 20 μl of 3-(4,5-dimethylthiazol-2-yl)-2,5-diphenyltetrazolium bromide (MTT) solution (Sigma-Aldrich, Merck KGaA, Darmstadt, Germany) at 37°C with 5% of CO_2_ for 4 h. Later, the culture medium was removed, and 150 μl of dimethyl sulfoxide was added. The plates were shaken in the dark for 10 min. The absorbance value at 490 nm was evaluated by the microplate reader (Bio-Rad Laboratories, Hercules, CA, United States).

### Flow Cytometry

After the coculture of NPCs and BMSCs, NPC suspensions (2 × 10^5^) were centrifuged at 200 *g* for 5 min, and the supernatant was removed. The cell precipitates were resuspended in 500 μl of the binding buffer using the Annexin V-fluorescein isothiocyanate (FITC) apoptosis detection kit I (556547, Becton, Dickinson and Company, Franklin Lakes, NJ, United States). The samples were mixed with 5 μl of Annexin V-FITC and propidium iodide and cultured for 15 min. The cell apoptosis was evaluated by the flow cytometer (FC500 MCL, Beckman Coulter, Chaska, MN, United States).

### Dual-Luciferase Reporter Gene Assay

The binding site of miR-101-3p and EIF4G2 was predicted by Starbase. The binding sequence and mutant sequence were cloned into the luciferase vector pGL3 (Pro-mega, Madison, WI, United States) to construct wild-type and mutant-type (EIF4G2-WT and EIF4G2-MUT) luciferase plasmids. The constructed plasmids were co-transfected with miR-101-3p mimic or mimic NC into 293T cells using Lipofectamine 2000. After 48 h, the relative luciferase activity was detected using the dual-lucy assay kit (Solarbio, Beijing, China).

### Statistical Analysis

Data analysis was analyzed and introduced using SPSS 21.0 (IBM Corp., Armonk, NY, United States) and GraphPad Prism 8.0 (GraphPad Software Inc., San Diego, CA, United States). First, the test of normality and homogeneity of variance was carried out, which showed that the data were consistent with normal distribution and homogeneity of variance. Data are expressed as mean ± SD. The independent sample *t*-test was adopted for comparison between two groups. One-way ANOVA was employed for the comparisons among multiple groups, following the Tukey’s multiple comparison test. The *p*-value was obtained from a two-tailed test, and *p* < 0.001 denoted the very statistical significance.

## Results

### Extracellular Matrix Degradation of NPCs in Patients With IDD

**FIGURE 1:**
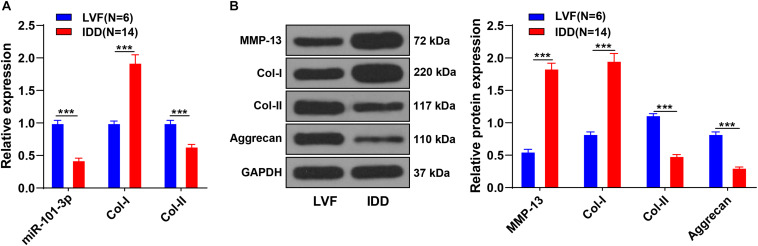
Extracellular matrix (ECM) degradation of nucleus pulposus cells (NPCs) in patients with intervertebral disk degeneration (IDD). NPCs were isolated from the intervertebral disk tissues of patients with IDD, with that of patients with LVF as control. **(A)** miR-101-3p, Collagen-I (Col-I), and Collagen-II (Col-II) expressions were detected using reverse transcription-quantitative polymerase chain reaction (RT-qPCR) and **(B)** MMP-13, Col-I/Col-II, and Aggrecan levels were detected using Western blotting. Data are presented as mean ± SD and analyzed using the independent sample *t*-test, ***p < 0.001.

The miR-101-3p expression is downregulated in NPCs of rats with IDD and associated with the levels of Col-I and Col-II (Gao Z.X. et al., 2020). To determine the function of miR-101-3p in IDD, we analyzed the expressions of miR-101-3p, Col-I, and Col-II in NPCs of 14 patients with IDD and 6 patients with LVF using the RT-qPCR. Compared with NPCs of patients with LVF, NPCs of patients with IDD showed downregulated miR-101-3p and Col-II expressions and upregulated Col-I expression (all *p* < 0.001; Figure 1A). Western blotting exhibited that NPCs of patients with IDD had upregulated MMP-13 and Col-I and downregulated Aggrecan and Col-II (*p* < 0.001; Figure 1B). These results suggested the involvement of miR-101-3p in IDD progression and the degradation of ECM of NPCs in patients with IDD.

### Bone Mesenchymal Stem Cells Promoted ECM Remodeling of Degenerated NPCs

**FIGURE 2:**
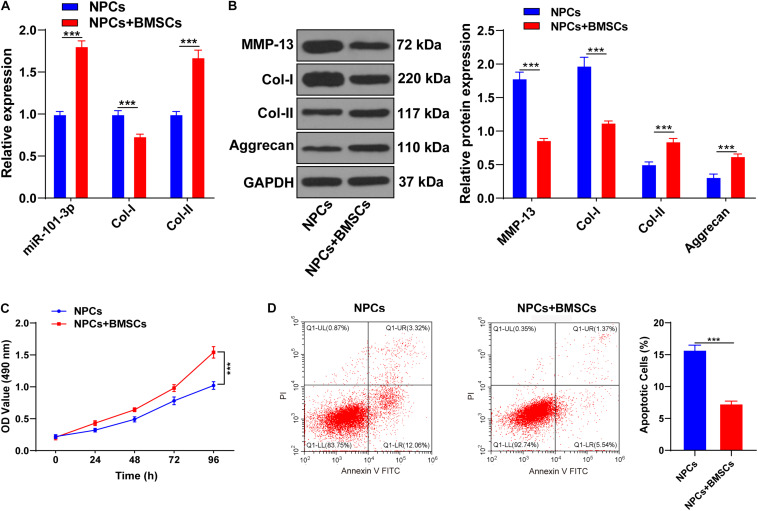
Bone mesenchymal stem cells (BMSCs) promoted ECM remodeling of NPCs. NPCs of patients with IDD were cocultured with BMSCs, with NPCs cultured alone as the control. **(A)** miR-101-3p, Col-I, and Col-II expressions were detected using the RT-qPCR; **(B)** MMP-13, Col-I, Col-II, and Aggrecan levels were detected using Western blotting; **(C,D)** the effects of BMSCs on the viability and apoptosis of NPCs were detected using MTT assay and flow cytometry. The cell experiments were repeated three times independently. Data are presented as mean ± SD. Data in panels **(A–C)** were analyzed using the independent sample *t*-test, ***p < 0.001.

Bone mesenchymal stem cells (BMSCs) can ameliorate IDD by attenuating the degeneration of NPCs (Li X. et al., 2019). NPCs of one patient with IDD were extracted for the *in vitro* study. BMSCs were cocultured with NPCs of one patient with IDD, and then expressions of miR-101-3p, Col-I, and Col-II were measured using the RT-qPCR. Compared with NPCs, NPCs cocultured with BMSCs showed increased Col-II and miR-101-3p and decreased Col-I (all *p* < 0.001; Figure 2A). Western blotting exhibited that compared with the NPCs, NPCs cocultured with BMSCs had downregulated MMP-13 and Col-I and upregulated Aggrecan and Col-II levels (*p* < 0.001; Figure 2B). MTT assay and flow cytometry revealed that compared with the NPCs, the viability of NPCs cocultured with BMSCs was enhanced, and the apoptosis rate was repressed (*p* < 0.001; Figures 2C,D). Taken together, BMSCs enhanced the viability, suppressed apoptosis, and facilitated ECM remodeling of degenerated NPCs. miR-101-3p expression in degenerated NPCs was notably upregulated after coculture of BMSCs and NPCs, which indicated that miR-101-3p was implicated in the process of BMSCs, promoting ECM remodeling of degenerated NPCs.

### Downregulation of miR-101-3p Inhibited the Effect of BMSCs on ECM Remodeling of Degenerated NPCs

**FIGURE 3:**
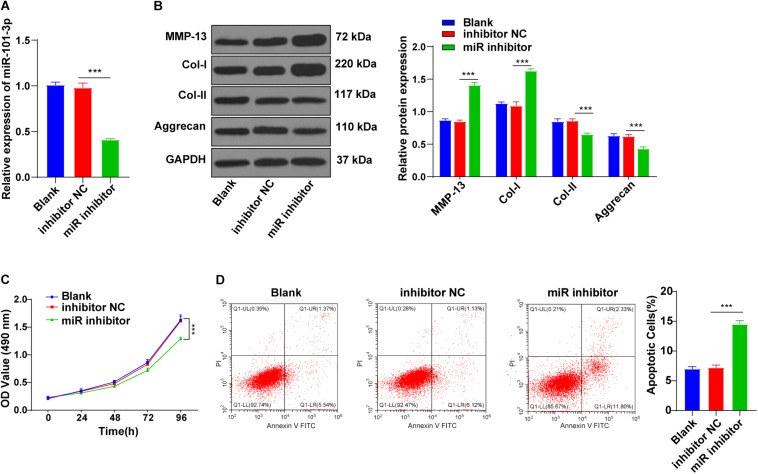
Downregulation of miR-101-3p inhibited the effect of BMSCs on ECM remodeling of NPCs. NPCs of patients with IDD were cocultured with BMSCs and then transfected with miR-101-3p inhibitor, with transfection of inhibitor negative control as control. **(A)** miR-101-3p expressions were detected using the RT-qPCR; **(B)** MMP-13, Col-I, Col-II, and Aggrecan levels were detected using Western blotting; and **(C,D)** viability and apoptosis of NPCs were detected using MTT assay and flow cytometry. The cell experiments were repeated three times independently. Data are presented as mean ± SD. Data were analyzed using one-way ANOVA, followed by the Tukey’s multiple comparison test, ***p < 0.001.

To explore the potential mechanism of miR-101-3p in BMSC-mediated IDD repair, the miR-101-3p inhibitor was transfected into the degenerated NPCs cocultured with BMSCs, with NPCs cocultured with BMSCs (blank group) and inhibitor NC as the control. miR-101-3p expression in miR-101-3p inhibitor-transfected NPCs was downregulated notably (*p* < 0.001; Figure 3A). Compared with the inhibitor NC group, the miR-101-3p inhibitor group showed promoted MMP-13 and Col-I expressions and decreased Aggrecan and Col-II expressions (all *p* < 0.001; Figure 3B). Additionally, compared with the inhibitor NC group, the miR-101-3p inhibitor group showed reduced cell viability (*p* < 0.001; Figure 3C) and enhanced apoptosis (*p* < 0.001; Figure 3D). In brief, the downregulation of miR-101-3p inhibited the effect of BMSCs on ECM remodeling of degenerated NPCs.

### miR-101-3p Targeted EIF4G2

**FIGURE 4:**
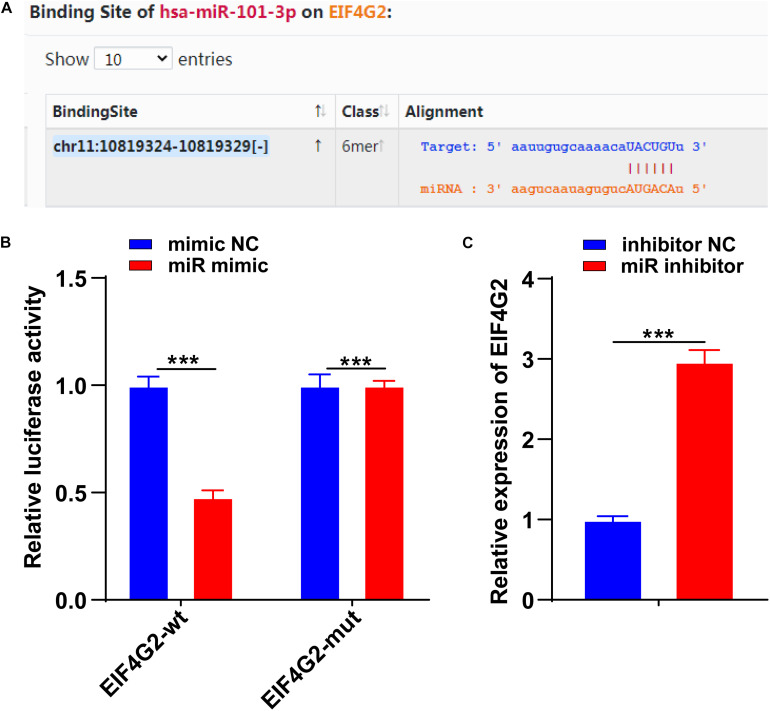
miR-101-3p targeted EIF4G2. **(A)** The binding site between miR-101-3p and EIF4G2 was predicted by using the Starbase database; **(B)** the binding relationship between miR-101-3p and EIF4G2 was verified using dual-luciferase reporter gene assay; and **(C)** expressions of EIF4G2 in miR-101-3p inhibitor-transfected NPCs were detected using the RT-qPCR. The cell experiments were repeated three times independently. Data are presented as mean ± SD. Data were analyzed using the independent sample *t*-test, ***p < 0.001.

To further determine the downstream target of miR-101-3p-mediated ECM remodeling, we conducted follow-up experiments. EIF4G2 is abnormally expressed in osteoarthritis (Gao S. et al., 2020). The binding site between miR-101-3p and EIF4G2 was predicted by using the Starbase database (Figure 4A). The binding relationship between miR-101-3p and EIF4G2 was verified using dual-luciferase reporter gene assay (*p* < 0.001; Figure 4B). Compared with the inhibitor NC group, EIF4G2 expression in the miR-101-3p inhibitor group was notably upregulated (*p* < 0.001; Figure 4C). miR-101-3p negatively regulated EIF4G2 expression.

### miR-101-3p Enhanced the Effect of BMSCs on ECM Remodeling of Degenerated NPCs by Targeting EIF4G2

**FIGURE 5:**
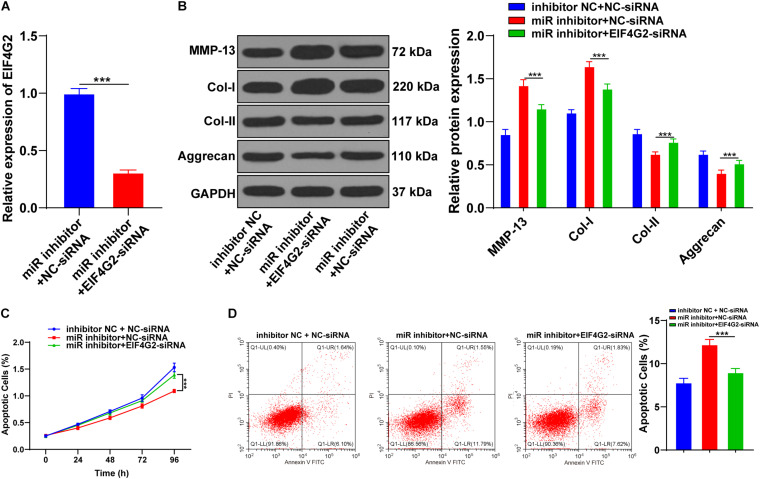
Downregulation of EIF4G2 reversed the inhibitory effect of miR-101-3p inhibitor on ECM remodeling of NPCs. **(A)** EIF4G2-siRNA transfection efficiency was confirmed using the RT-qPCR; **(B)** MMP-13, Col-I, Col-II, and Aggrecan levels were detected using Western blotting; **(C,D)** viability and apoptosis of NPCs were detected using MTT assay and flow cytometry. The cell experiments were repeated three times independently. Data are presented as mean ± SD. Data in panel **(A)** were analyzed using the independent sample *t*-test; and data in panels **(B–D)** were analyzed using one-way ANOVA, followed by the Tukey’s multiple comparison test, ***p < 0.001.

Functional rescue experiments were performed to verify whether miR-101-3p enhanced the promoting effect of BMSCs on ECM remodeling of degenerated NPCs by targeting EIF4G2. BMSC-cocultured NPCs were transfected with EIF4G2-siRNA, and the transfection efficiency was confirmed using the RT-qPCR (*p* < 0.001; Figure 5A). Downregulation of EIF4G2 reversed miR-101-3p inhibitor-mediated ECM protein levels in NPCs (*p* < 0.001; Figure 5B), as well as the viability and apoptosis of NPCs (*p* < 0.001; Figures 5C,D). miR-101-3p was confirmed to enhance the promoting effect of BMSCs on ECM remodeling of NPCs by targeting EIF4G2.

## Discussion

Intervertebral disk degeneration (IDD) is commonly caused by excessive degeneration of ECM, increased death of IVD cells, and impairment of biomechanical functions (Johnson et al., 2015). Transplantation of BMSCs can retard or reverse diseased IVD (Cao et al., 2015). Specific regulation of miRs also represents a promising approach for the management of IDD (Li et al., 2015). This study clarified that miR-101-3p enhanced the effect of BMSCs on ECM remodeling in IDD.

The pathogenesis of IDD concerns the degeneration of the water-binding ECM, mechanical overloading, and catabolic cell response (Vergroesen et al., 2015). With the degeneration of ECM, Col-II expression is gradually reduced, while Col-I expression is promoted (Vergroesen et al., 2015). Aggrecan represents the main non-collagenous component of ECM, which endows IVD with the ability to bear compressive load (Zhang et al., 2018). MMP-13 constitutes one of the primary catabolic factors in the ECM metabolism of NPCs (Wang et al., 2019). Patients with IDD showed elevated MMP-13 and Col-I expressions and reduced Col-II and Aggrecan expressions. Elevated MMP-13 expression is concerned with ECM degeneration (Liu et al., 2019), and the loss of Aggrecan causes the impairment of ECM function and initiation of IDD (Kepler et al., 2013). Deregulated miRs in NPCs functionally participate in IDD progression by acting on NPC proliferation, apoptosis, and ECM synthesis/degradation (Li Z. et al., 2019). miR-101 participates in ECM degradation in osteoarthritis (Dai et al., 2015; Lu et al., 2020). Still, the effect of miR-101-3p on ECM degradation of NPCs remains unclear. We showed that miR-101-3p expression was reduced in patients with IDD. Gao Z.X. et al. (2020) have unveiled that miR-101-3p is downregulated in degenerated NPCs, and miR-101-3p participates in the modulation of NPC proliferation and apoptosis. In brief, miR-101-3p is implicated in IDD progression.

Bone mesenchymal stem cells (BMSCs) are crucial tools for tissue repair, which can differentiate into NP-like cells in IVD and promote the proteoglycan content of ECM (Xu et al., 2016). Therefore, BMSCs were cocultured with NPCs of a patient with IDD. The BMSC-cocultured NPCs showed enhanced Aggrecan, Col-II, and miR-101-3p expressions and decreased Col-I and MMP-13 expressions. NPCs, the major cell type residing in NP, are responsible for the integrity of IVD *via* the role of producing ECM components (Wang et al., 2014; Li et al., 2015; Cheng et al., 2018). Deregulated phenotypes of NPCs, including aberrant apoptosis, autophagy, and proliferation, contribute to the initiation and progression of IDD (Li et al., 2018). The reduction of NPCs and the subsequent degeneration of ECM contribute to the progression of IDD (Makino et al., 2017; Yang et al., 2018). Coculture of NPCs with BMSCs can notably repress the degeneration and apoptosis of NPCs (Hu et al., 2015). Consistently, we revealed that NPCs cocultured with BMSCs had enhanced NPC viability and suppressed apoptosis. Yi et al. (2014) have clarified that BMSC transplantation enhances the ECM content by repressing ECM degradation and facilitating ECM synthesis. BMSCs possess an anti-apoptotic effect on IDD through the mitochondrial apoptotic pathway in AF cells (Hu et al., 2017). Taken together, BMSCs repressed NPC apoptosis and facilitated ECM remodeling of NPCs.

Additionally, miR-101-3p expression in NPCs was notably upregulated after coculture, which indicated that miR-101-3p was involved in the process of BMSCs, promoting ECM remodeling of NPCs. The promotion function of miR-101 on chondrogenic differentiation of BMSCs has been revealed previously (Gao et al., 2019). Then, we transfected miR-101-3p inhibitor into BMSC-cocultured NPCs. miR-101-3p inhibitor-transfected NPCs showed promoted MMP-13 and Col-I expressions and decreased Aggrecan and Col-II expressions. Additionally, miR-101-3p inhibitor-transfected NPCs showed reduced cell viability and enhanced apoptosis. In brief, the downregulation of miR-101-3p inhibited the effect of BMSCs on ECM remodeling of NPCs.

Thereafter, we further determined the downstream target of miR-101-3p. The binding site between miR-101-3p and EIF4G2 was predicted by using the Starbase database. Translation of most mRNAs is modulated at the initiation level, a process demanding the protein complex called EIF4F (including EIF4E, EIF4G, and EIF4A) (Caron et al., 2004; Xie et al., 2017). EIF4G has two functional homologs in mammals, namely, EIF4G1 and EIF4G2 (Caron et al., 2004). EIF4G2 expression is promoted in osteoarthritis cartilage tissues, and EIF4G2 may be implicated in the pathogenesis of osteoarthritis (Gao S. et al., 2020). Nevertheless, the role of EIF4G2 in IDD remained unknown, and this study filled this gap to a certain extent. EIF4G2 expression of miR-101-3p inhibitor-transfected NPCs was notably upregulated, indicating that miR-101-3p negatively regulated EIF4G2 expression. The functional rescue experiments were performed. BMSC-cocultured NPCs were transfected with EIF4G2-siRNA, and the results exhibited that the downregulation of EIF4G2 reversed miR-101-3p inhibitor-mediated ECM protein levels in NPCs, as well as viability and apoptosis of NPCs. It was confirmed that miR-101-3p enhanced the promoting effect of BMSCs on ECM remodeling of NPCs by targeting EIF4G2.

To sum up, miR-101-3p increased the promoting effect of BMSCs on ECM remodeling of degenerated NPCs by targeting EIF4G2. This study unveiled a new mechanism of BMSCs in the treatment of IDD. However, there are still some limitations and deficiencies in this study. In this study, we chose the commercial single mesenchymal stem cell (MSC) case to study and ignored the variation among individuals, which led to the oversimplification of the conclusion. Additionally, we only isolated and cultured NPCs from one patient with IDD for the *in vitro* study. Whether this regulatory mechanism is different in degenerated NPCs from patients with IDD with different clinical parameters, different clinical grading, and different genders remains to be further explored. This study did not explain what substances MSCs may secrete to drive the increase of miR-101-3p. The existing studies have demonstrated that MSC-derived exosomes can repair IDD by carrying miRNAs (Zhang et al., 2020; Zhu et al., 2020). Whether the increase of miR-101-3p was driven by MSC-derived exosomes remained unidentified. In this study, we selected degenerated NPCs from patients with IDD but did not study the effect of miR-101-3p on non-degenerated NPCs from patients with LVF. Whether the decrease of miR-101-3p can drive the catabolic phenotype of NPCs is also what we need to continue to explore. In addition, the mechanism of BMSCs promoting ECM remodeling *via* miR-101-3p/EIF4G2 axis also needs further functional experimental verification *in vivo*. In the future, we will continue to determine that whether MSCs can promote the ECM remodeling of degenerated NPCs by secreting exosomes to drive the increase of miR-101-3p and conduct functional experiments *in vivo*.

## Data Availability Statement

The original contributions presented in the study are included in the article/supplementary material, further inquiries can be directed to the corresponding author/s.

## Ethics Statement

The studies involving human participants were reviewed and approved by the Ethical Committee of The Second Affiliated Hospital of Shandong First Medical University. The patients/participants provided their written informed consent to participate in this study. The animal study was reviewed and approved by the Ethical Committee of Shandong First Medical University.

## Author Contributions

ZW: conceptualization and methodology. XD: data curation and writing—original draft preparation. FC: investigation. XZ: supervision and validation. JW: writing—reviewing and editing. All authors contributed to the article and approved the submitted version.

## Conflict of Interest

The authors declare that the research was conducted in the absence of any commercial or financial relationships that could be construed as a potential conflict of interest.

## Publisher’s Note

All claims expressed in this article are solely those of the authors and do not necessarily represent those of their affiliated organizations, or those of the publisher, the editors and the reviewers. Any product that may be evaluated in this article, or claim that may be made by its manufacturer, is not guaranteed or endorsed by the publisher.
